# Frontal Sinus Epidermoid Cyst: A Rare Clinical Entity With Diagnostic Challenges and Surgical Considerations—A Case Report

**DOI:** 10.1002/ccr3.70887

**Published:** 2025-09-15

**Authors:** Mohammadreza Firouzifar, Sahar Shirkhani, Hamed Pourkhosravi, Melika Karimi

**Affiliations:** ^1^ Department of Otolaryngology—Head and Neck Surgery Amir‐Alam Hospital, Tehran University of Medical Sciences Tehran Iran

**Keywords:** benign cystic lesion, bone erosion, endoscopic sinus surgery, ENT case report, epidermoid cyst, frontal sinus, intraorbital extention, orbit, rare sinonasal tumor, skull base lesion

## Abstract

Epidermoid cysts are rare lesions in the frontal sinus, often presenting with nonspecific symptoms such as headaches and localized swelling. These factors can complicate diagnosis and management. This report describes an epidermoid cyst that was initially suspected to be a malignant mass due to the erosion of the anterior wall and the sclerotic nature of the lateral wall, which had extended toward the orbit. This conclusion is supported by the observation that its pressure has pushed the eye downward, suggesting a primary origin in the frontal sinus rather than the orbit. We present a case of a 21‐year‐old woman who had a 2‐year history of right periorbital swelling and intermittent headaches. Imaging studies revealed a lesion in the right frontal sinus, and surgical intervention resulted in the excision of the cyst. During surgery, after removing the bone shell, it was confirmed that the lesion was an epidermoid cyst, ruling out the initial suspicion of malignancy. Histopathological examination confirmed the diagnosis of an epidermoid cyst. This case underscores the importance of imaging studies in diagnosis and highlights the effectiveness of surgical intervention for managing symptomatic epidermoid cysts in the frontal sinus. We aim to investigate how much of the normal anatomy of the frontal sinus and periorbital structures may have been affected, and how symptoms were managed.

**Summary**

- Epidermoid cysts of the frontal sinus are rare and may radiologically mimic malignancies due to bone erosion and orbital displacement.
- This case underscores the diagnostic value of advanced imaging and highlights the efficacy of endoscopic surgical excision in achieving complete resolution without recurrence.

## 1 Introduction

Epidermoid cysts are benign, slow‐growing lesions originating from ectodermal cells and are commonly found in various anatomical locations, including the skin, central nervous system, and paranasal sinuses [1, 2]. The occurrence of epidermoid cysts within the sinonasal region is particularly rare, with their incidence estimated to account for less than 1% of all sinonasal neoplasms. This rarity can lead to delays in diagnosis due to a lack of awareness among healthcare providers [3, 4]. Delays in diagnosing a progressive cystic lesion that can exert pressure on adjacent structures, such as the orbit and brain, can lead to increased complications both before and during surgery. To address this issue, we have established careful collaboration among specialists from various fields to minimize surgical complications and improve diagnostic accuracy.

Patients presenting with frontal sinus epidermoid cysts typically exhibit nonspecific symptoms, such as persistent headaches, facial discomfort, and localized swelling in the periorbital area [5, 6].

Furthermore, complications become clinically significant when the cyst enlarges and exerts mass effect on surrounding anatomical structures. In our case, this was evident by erosion of the anterior wall of the frontal sinus and sclerosis of the lateral wall, which had extended toward the orbital cavity. Such complications may lead to mucocele formation, orbital cellulitis, or, in rare cases, even intracranial extension, as emphasized in several studies that underline the potential for severe outcomes in untreated cases [7, 8].

Recent literature has highlighted the importance of not overlooking the differential diagnosis of an epidermoid cyst, which may masquerade as more benign conditions, such as frontal sinusitis. For instance, the case study by Abedi et al. reported a patient whose symptoms of frontal headache were mistakenly attributed to frontal sinusitis for over a decade [9]. As noted by Min et al., the need to monitor for complications such as Pott's puffy tumor is crucial in similar cases that may have a secondary inflammatory picture [10]. In the maxillary sinus, it is important to consider other differential diagnoses, such as ectopic teeth. However, there is a wide range of conditions that can mimic the appearance of ectopic teeth, including rhinoliths, sinoliths, compound or complex odontomas, and calcifying odontogenic cysts. Each of these conditions has unique clinical and radiological characteristics that can help differentiate them from an ectopic tooth [11]. Some maxillofacial tumors and cysts may be asymptomatic or present with symptoms similar to other diseases. Therefore, proper management is crucial to ensure that these important diagnoses are not overlooked. The timing of symptom onset is critical in recognizing potential complications. Recurrent or progressively severe symptoms may indicate underlying pathological changes, highlighting the need for immediate and comprehensive evaluation. Accurate imaging techniques, particularly computed tomography (CT) and magnetic resonance imaging (MRI), played an indispensable role in differentiating the cyst from potential malignancies by revealing the extent of the sclerotic changes and erosion associated with the lesion [4, 12, 13].

Epidemiologically, although these cysts can occur across various age groups, they are mostly reported in young adults. While the exact causes of their development are not fully understood, they may be related to developmental issues or past injuries [2, 3, 4]. This is further supported by Samdani et al. and Toktaş et al., who highlight cases with traumatic histories leading to cyst formations and notable complications like hydrocephalus and encephaloceles [14, 15].

Several studies have highlighted the presence of epidermoid cysts in the periorbital region and frontal sinus. Literature, including research by Morotomi et al., emphasizes that these lesions can lead to the progressive destruction of nearby anatomical structures, potentially resulting in complications such as diplopia [5].

This report discusses the clinical path of a 21‐year‐old woman diagnosed with an epidermoid cyst thought to have started in the frontal sinus, as indicated by her symptoms of ongoing swelling around the right eye. This case emphasizes the need for a team approach to evaluation and treatment. Surgical removal of the cyst is essential, along with managing any complications that may arise during recovery. Proper follow‐up is crucial to monitor for recurrence and manage any long‐term effects.

This report aims to raise awareness about epidermoid cysts in the frontal sinus and the importance of early diagnosis and effective management to reduce complications and improve patient outcomes.

## 2 Case Presentation

### 2.1 Patient Characteristics

A 21‐year‐old female with no significant past medical history presented to the clinic with a two‐year history of intermittent swelling in the right periorbital region. She reported that the swelling fluctuated in size, occasionally increasing in diameter, and was accompanied by periodic headaches. There was no history of trauma, infection, or recent sinusitis. Importantly, she denied any associated symptoms such as visual disturbances, diplopia, photophobia, or changes in ocular mobility, which could indicate more serious underlying pathology.

### 2.2 Clinical Findings

On physical examination, a moderate, non‐tender swelling measuring approximately 2 cm in diameter was noted in the right periorbital area. The lesion was soft to palpation, with no signs of erythema or induration. No palpable cervical lymphadenopathy was appreciated. A comprehensive neurological evaluation revealed no focal deficits, cranial nerve abnormalities, or other signs of neurological compromise. Routine laboratory tests, including complete blood count, comprehensive metabolic panel, and additional inflammatory markers, returned within normal limits, further supporting a benign process.

### 2.3 Clinical Course and Management

#### 2.3.1 Imaging Studies

**FIGURE 1:**
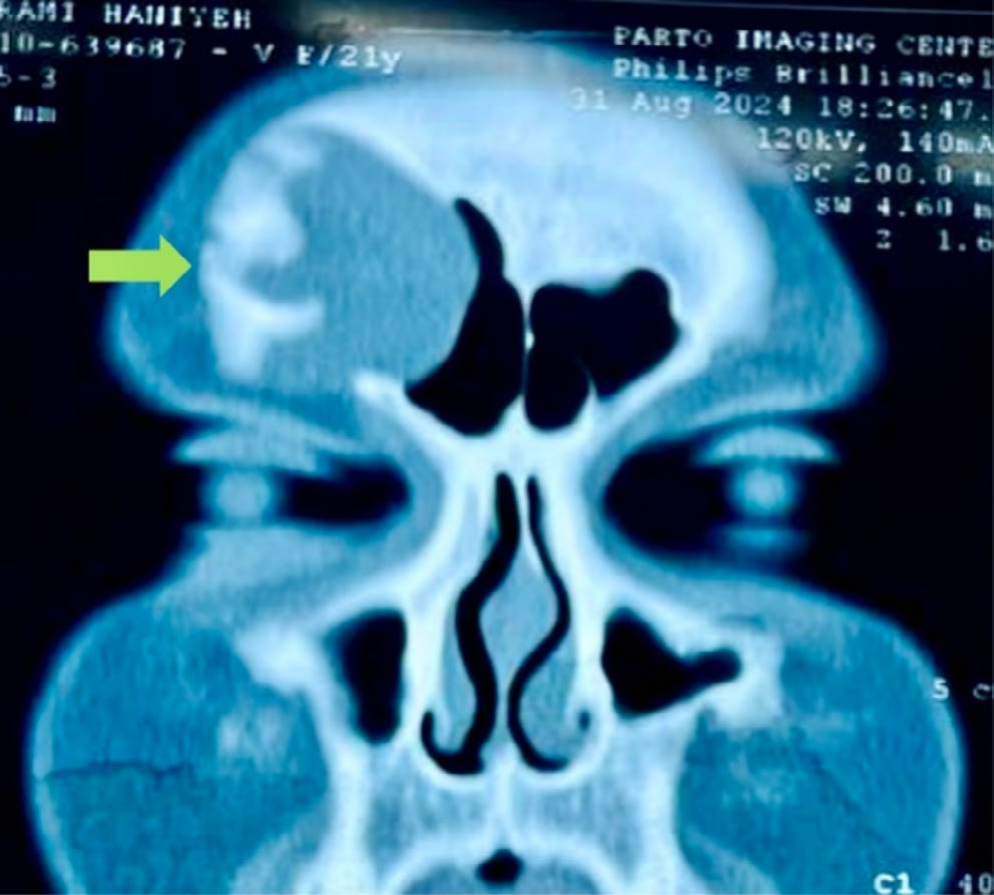
Coronal CT scan showing a hypodense mass occupying the right frontal sinus, with expansion and erosion of the anterior sinus wall.

**FIGURE 2:**
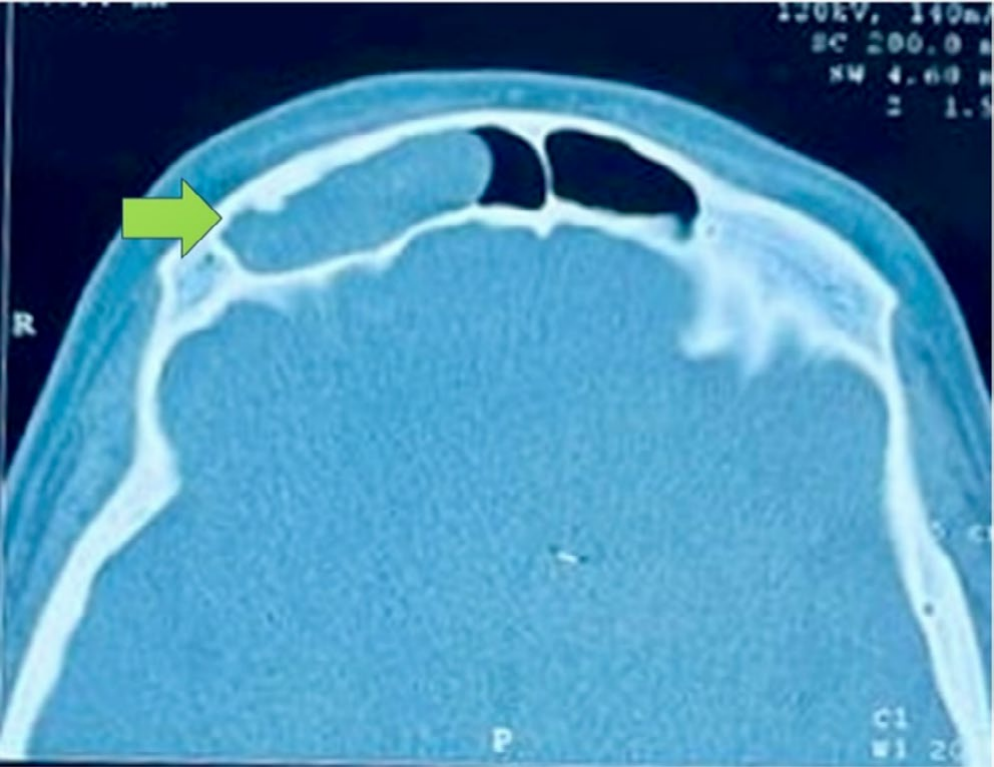
Axial CT image demonstrating thinning and remodeling of the anterior table of the right frontal sinus.

**FIGURE 3:**
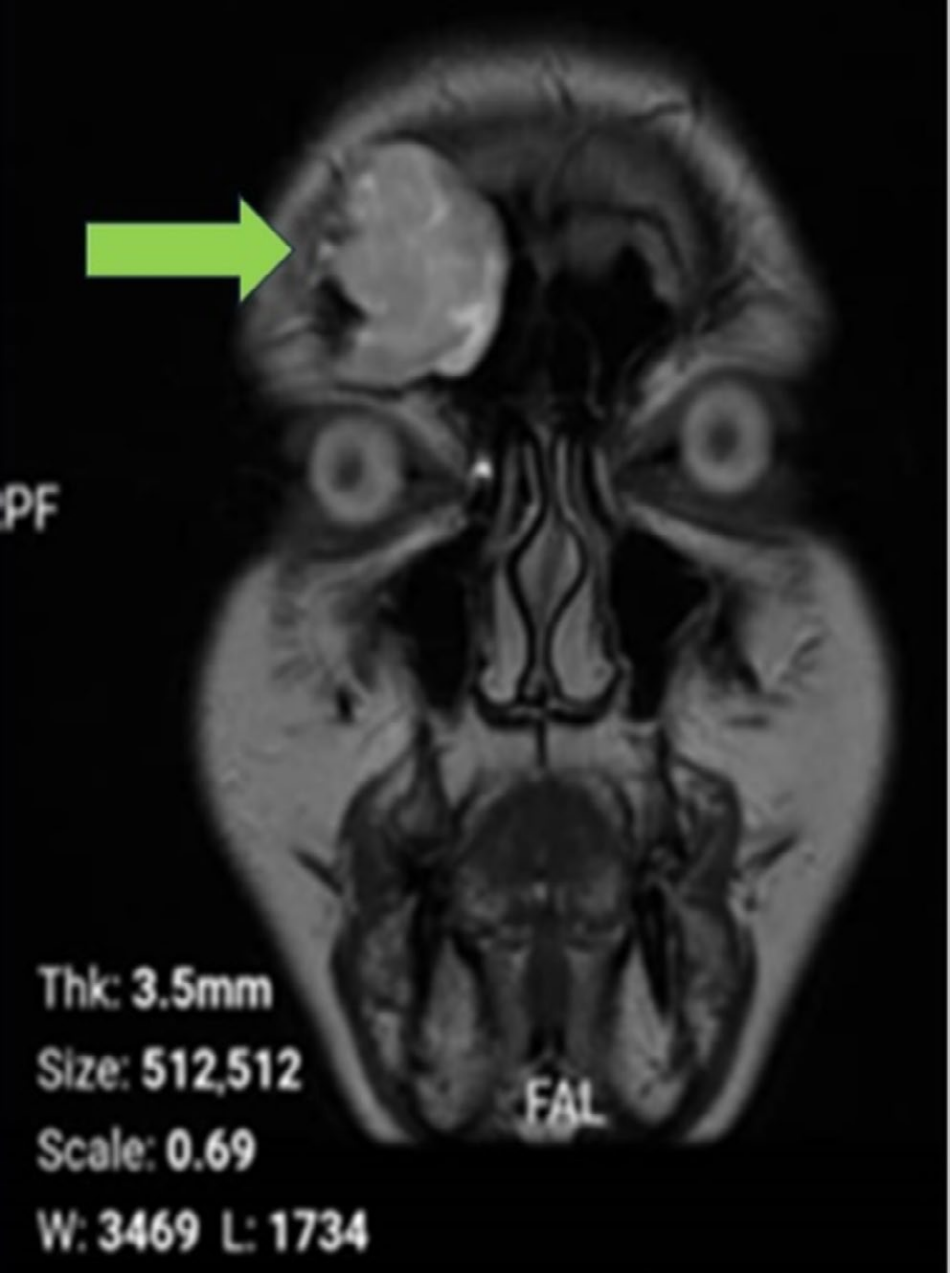
Coronal T2‐weighted MRI revealing a intermediate to mildly hyperintense lesion in the frontal sinus with inferior displacement of surrounding tissues.

**FIGURE 4:**
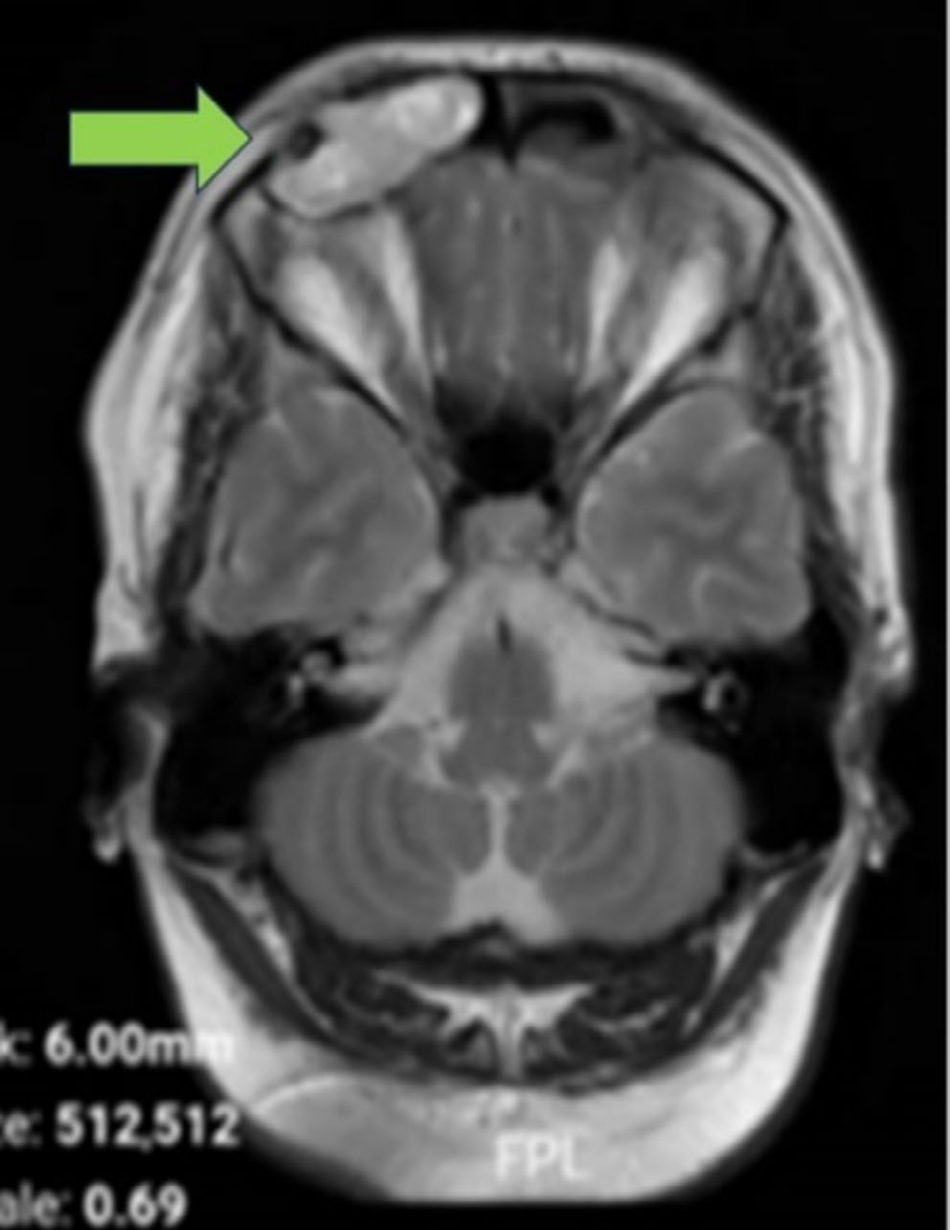
Axial T2‐weighted MRI showing a intermediate to mildly hyperintense lesion within the right frontal sinus, suggesting a cystic structure.

**FIGURE 5:**
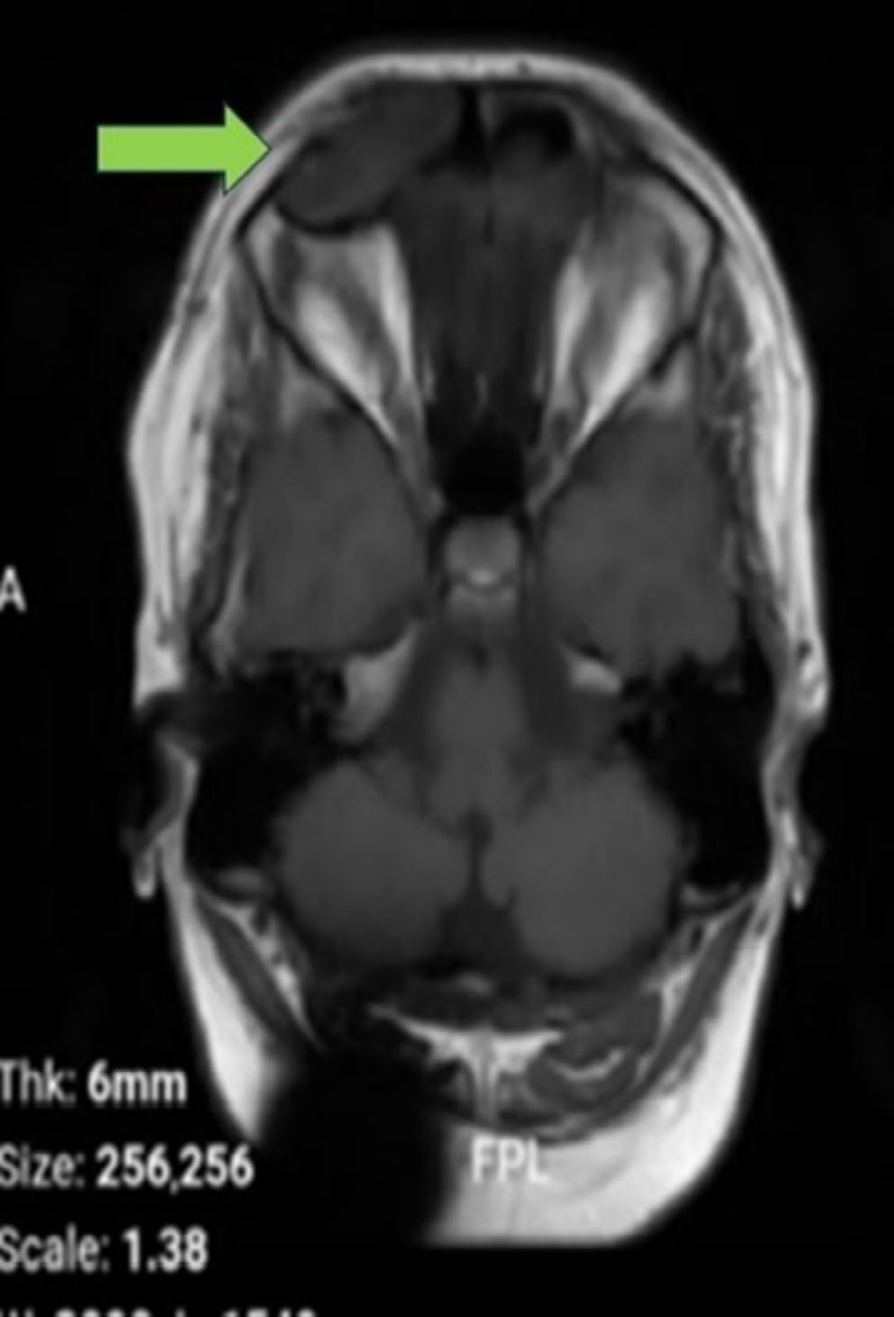
Axial gadolinium‐enhanced T1 MRI confirming non‐enhanced lesion with mass effect on the right orbit causing displacement.

**FIGURE 6:**
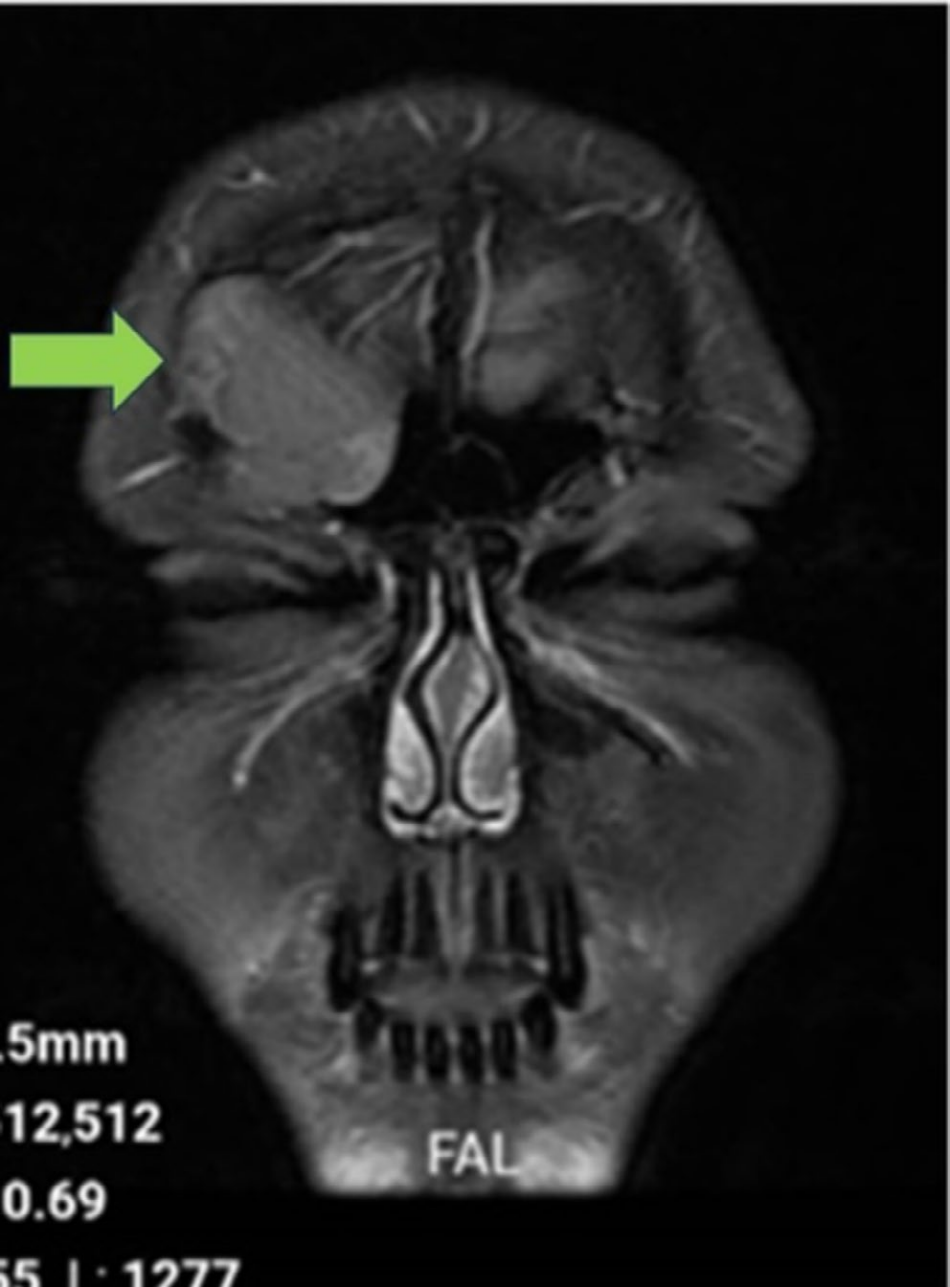
Coronal gadolinium‐enhanced T1‐weighted MRI showing absence of enhancement, indicating a non‐vascular lesion consistent with an epidermoid cyst.

To further assess the periorbital mass, the patient underwent both CT and MRI of the brain and sinuses. A CT scan was obtained to identify potential bony erosions or complications associated with the cyst (Figure S1; Figure 1). MRI delineated the extent of the cyst and its relationship to surrounding soft tissues and vascular structures. The imaging studies demonstrated a well‐defined lesion in the right frontal sinus, measuring approximately 15 × 30 mm. The lesion exhibited hypodense characteristics on CT (Figure S2; Figure 2) and demonstrated hyperintense signals on T2‐weighted MRI sequences (Figures S3, S4, S7 and S8; Figures 3 and 4), with a hypointense rim noted on T1‐weighted images (Figures S5 and S6; Figures 5 and 6). These imaging studies confirmed the erosion of the anterior wall of the frontal sinus and sclerosis of the lateral wall, thus raising initial concerns for a malignant mass. This also indicated that the pressure had caused the eye to be pushed downward. The imaging also ruled out other potential causes, such as neoplasms or acute inflammatory processes (Figures 1, 2, 3, 4, 5, 6; Figures S1–S8).

#### 2.3.2 Surgical Intervention

Given the lesion's persistent nature and associated symptoms, surgical intervention was planned. The patient underwent endoscopic frontal sinus surgery under general anesthesia. Utilizing 0° and 30° endoscopes, we performed a meticulous excision of the lesion beginning with access through the frontal sinus ostium. During the procedure, specific endoscopic tools such as microdebriders and suction devices were employed to enhance visualization and enable precise dissection. The surgical technique involved careful dissection to ensure the integrity of surrounding anatomical structures while achieving complete drainage of the cyst contents. In line with the techniques described by Chandra et al., we emphasize minimizing trauma to adjacent tissues to reduce the risk of complications [16]. Intraoperative frozen section analysis was performed to confirm that the lesion was an epidermoid cyst after removing the bony shell, ending the preliminary concerns regarding malignancy.

#### 2.3.3 Postoperative Management

After the surgery, the patient was watched in the recovery room for possible complications. The patient had a smooth recovery and was discharged on the same day as her surgery. She was given specific instructions on managing pain and identifying signs of possible complications, such as swelling or fever. She was advised to return for follow‐ups to assess the frontal sinus function and watch for recurrence.

During follow‐up visits, we established careful monitoring protocols, including further imaging if any concerning symptoms arose, to quickly address complications or potential recurrence.

Subsequent evaluations at 2 weeks, 1 month, 2 months, and 6 months postoperatively showed significant improvement in the symptoms. Complete resolution of the swelling around her eye was noted, with no signs of blockage or recurrence in the frontal sinus. Importantly, she reported substantial relief from her previous headaches, which positively affected her daily activities and overall quality of life. These results emphasize the importance of follow‐up and show the effectiveness of the surgical procedure. Our treatment plan for this rare condition confirms its primary origin from the frontal sinus, highlighting the significance of thorough assessment and surgical care.

## 3 Discussion

Epidermoid cysts are often asymptomatic but can cause significant issues when they become large enough to press on nearby structures around the eye or in the frontal sinus. In our case, the patient had swelling around her eye and headaches, likely because the cyst was expanding and pushing on the surrounding tissues. Notably, the anterior wall was damaged, and the lateral wall had hardened, which raised initial concerns about cancer. In addition to the CT scan, we performed an MRI on the patient, which showed that the cause of the bone erosion was a cyst with a compressive effect, but even so, we had prepared ourselves in the operating room for cases such as cancer. As the pressure increases, it can affect blood flow or irritate nerves, exacerbating symptoms.

This case highlights the need for proper imaging to get an accurate diagnosis. The size and location of epidermoid cysts can look similar to other conditions, like dermoid cysts or lipomas. The imaging findings in this case were crucial for determining the diagnosis, highlighting the importance of thorough imaging practices in sinonasal conditions [12, 13]. Although our patient experienced notable pressure on the ocular structures, she did not present with visual disturbances or diplopia, showcasing a unique aspect of this case.

The rarity of epidermoid cysts in the frontal sinus necessitates a high index of suspicion, particularly in young adults presenting with periorbital swelling. Prompt intervention is essential; recurrent or progressively severe symptoms may indicate significant underlying changes that require immediate evaluation. The evolution of the symptoms further underlines the importance of timely intervention. Given that other documented cases have shown how traumatic or inflammatory processes related to cysts can lead to complications such as encephaloceles and hydrocephalus, our findings emphasize the need for vigilant monitoring [14, 15].

The pressure from the cyst has caused the eye to be pushed downward, which is indicative of the significant structural changes observed in imaging. An accurate diagnosis is critical, as misdiagnosing these cysts can lead to unnecessary procedures and potentially worsen patient outcomes. In this case, successful surgical intervention, coupled with the patient's smooth postoperative recovery, underscores the effectiveness of endoscopic techniques in managing symptomatic lesions of this nature. This aligns with the findings of Chandra et al., who demonstrated successful endoscopic management of epidermoid cysts in paranasal sinuses, although our case was confined to the frontal sinus [16]. Complications can arise when a cyst increases in size, exerting pressure on nearby structures or resulting in a secondary infection, as shown in our patient's imaging results. These complications may lead to the formation of a mucocele, orbital cellulitis, or, in rare cases, even extend to the intracranial area. This underscores the importance of timely intervention [17, 18].

Comprehensive postoperative management plans that include monitoring for recurrence are essential. Addressing lingering symptoms that may affect a patient's quality of life can also be beneficial, and it may include pain management strategies and physical therapy to alleviate discomfort or lingering headaches. Endoscopic approaches allow for direct visualization and targeted access to the lesion while minimizing disruption to surrounding tissues, which is especially beneficial in delicate areas like the frontal sinus and orbit [5, 7].

Furthermore, this case reinforces the necessity for interdisciplinary collaboration in managing such lesions, integrating insights from radiology, surgery, and pathology to optimize patient outcomes. Continuous research into the long‐term management, potential recurrence, and outcomes following surgical intervention for epidermoid cysts is warranted to refine treatment protocols further. Increasing awareness among clinicians regarding the diverse presentations of sinonasal cysts is vital for facilitating timely diagnosis and treatment [2, 3].

## 4 Conclusion

This case report highlights the successful diagnosis and management of an epidermoid cyst located in the frontal sinus of a young female patient. Some authors suggest that the cause of these lesions may be acquired, often as a result of penetrating skull trauma that allows cutaneous tissues to invade the diplopia [19].

Initial imaging findings raised concerns for a malignant mass due to the erosion and sclerotic changes in the sinus walls, but subsequent surgical findings confirmed that it was an epidermoid cyst. Imaging studies proved critical in guiding diagnosis, while endoscopic surgical techniques facilitated the effective cyst removal with minimal complications. The successful outcome of this case underscores the necessity for clinicians to maintain a high index of suspicion for rare entities like epidermoid cysts in patients presenting with nonspecific periorbital symptoms.

Importantly, ongoing follow‐up is essential to monitor for recurrence and postoperative complications, ensuring the continued efficacy of the intervention. Healthcare providers should be aware of the similarities between symptoms of epidermoid cysts and more common conditions like sinusitis, as timely recognition and intervention can significantly improve patient outcomes. This case heightens clinicians' awareness regarding the presenting features and potential management strategies for similar lesions. Research indicates that treatment typically involves surgical excision to alleviate symptoms and minimize the risk of recurrence. This approach is supported by the findings of Pagkou et al., Kumaran et al., and Law et al., who demonstrated that complete resection is effective in reducing the likelihood of recurrence. We hope to improve early diagnosis, optimize treatments, and enhance overall patient care by promoting knowledge about epidermoid cysts in the frontal sinus.

## Author Contributions

**Mohammadreza Firouzifar:** conceptualization, supervision, writing – review and editing. **Sahar Shirkhani:** investigation, writing – original draft. **Hamed Pourkhosravi:** investigation, writing – original draft. **Melika Karimi:** investigation, project administration, writing – original draft.

## Consent

Written informed consent was obtained from the patient for publication of this case report and accompanying images.

## Conflicts of Interest

The authors declare no conflicts of interest.

## Supporting information


**Figure S1:** Coronal CT view showing preserved bony margins with sclerotic changes around the lesion, consistent with a chronic slow‐growing process.
**Figure S2:** Axial CT image illustrating posterior extension of the lesion within the frontal sinus.
**Figure S3:** Axial T2‐weighted MRI image showing further extension of the lesion posteriorly.
**Figure S4:** Axial T1‐weighted MRI showing the lesion as slightly hyperintense compared to brain parenchyma.
**Figure S5:** Axial post‐contrast T1‐weighted MRI with gadolinium demonstrating no significant enhancement, consistent with a benign non‐vascular lesion.
**Figure S6:** Axial gadolinium‐enhanced T1 MRI confirming absence of enhancement, supporting the diagnosis of an epidermoid cyst.
**Figure S7:** Coronal T2 MRI showing detailed margins of the lesion and its relationship with the adjacent orbit.
**Figure S8:** Coronal T2‐weighted MRI highlighting expansion of the right frontal sinus with thinning of the superior orbital wall.

## Data Availability

The data supporting the findings of this case report are not available due to privacy and confidentiality restrictions.
