# Supplementary material for: Frontal Sinus Epidermoid Cyst: A Rare Clinical Entity With Diagnostic Challenges and Surgical Considerations—A Case Report
Source: Clin Case Rep. 2025 Sep 15;13(9):e70887. doi: 10.1002/ccr3.70887 (PMC12436175; doi:10.1002/ccr3.70887)
Supplement: Supplementary file 1 — Figure S1: Coronal CT view showing preserved bony margins with sclerotic changes around the lesion, consistent with a chronic slow‐growing process. Figure S2: Axial CT image illustrating posterior extension of the lesion within the frontal sinus. Figure S3: Axial T2‐weighted MRI image showing further extension of the lesion posteriorly. Figure S4: Axial T1‐weighted MRI showing the lesion as slightly hyperintense compared to brain parenchyma. Figure S5: Axial post‐contrast T1‐weighted MRI with gadolinium demonstrating no significant enhancement, consistent with a benign non‐vascular lesion. Figure S6: Axial gadolinium‐enhanced T1 MRI confirming absence of enhancement, supporting the diagnosis of an epidermoid cyst. Figure S7: Coronal T2 MRI showing detailed margins of the lesion and its relationship with the adjacent orbit. Figure S8: Coronal T2‐weighted MRI highlighting expansion of the right frontal sinus with thinning of the superior orbital wall. [file CCR3-13-e70887-s001.zip › ccr370887-sup-0001-supplamantary figures.docx]

**Supplementary figures legend:**

Supplementary Figure 1.

Coronal CT view showing preserved bony margins with sclerotic changes around the lesion, consistent with a chronic slow-growing process.

Supplementary Figure 2.

Axial CT image illustrating posterior extension of the lesion within the frontal sinus.

Supplementary Figure 3.

Axial T2-weighted MRI image showing further extension of the lesion posteriorly.

Supplementary Figure 4.

Axial T1-weighted MRI showing the lesion as slightly hyperintense compared

to brain parenchyma.

Supplementary Figure 5.

Axial post-contrast T1-weighted MRI with gadolinium demonstrating no significant

enhancement, consistent with a benign non-vascular lesion.

Supplementary Figure 6.

Axial gadolinium-enhanced T1 MRI confirming absence of enhancement, supporting the diagnosis of an epidermoid cyst.

Supplementary Figure 7.

Coronal T2 MRI showing detailed margins of the lesion and its relationship with the

adjacent orbit.

Supplementary Figure 8.

Coronal T2-weighted MRI highlighting expansion of the right frontal sinus with thinning of the superior orbital wall.
