# Supplementary figures and images for: Frontal Sinus Epidermoid Cyst: A Rare Clinical Entity With Diagnostic Challenges and Surgical Considerations—A Case Report
Source: Clin Case Rep. 2025 Sep 15;13(9):e70887. doi: 10.1002/ccr3.70887 (PMC12436175; doi:10.1002/ccr3.70887)

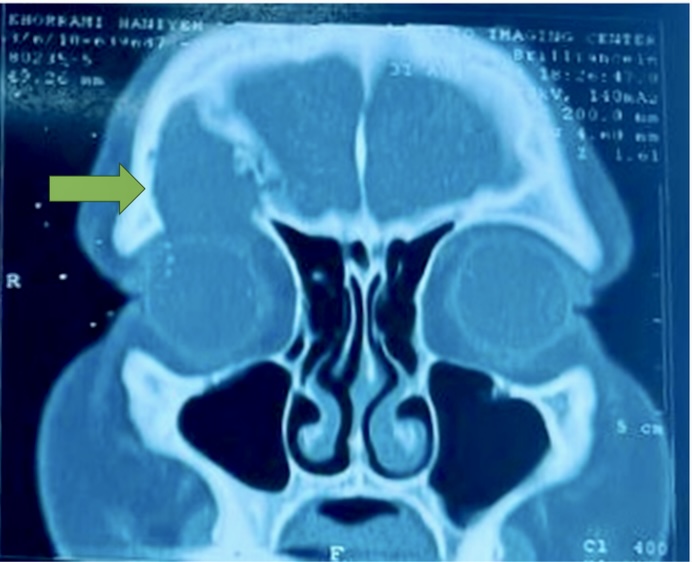

Supplement: Supplementary file 1 — Figure S1: Coronal CT view showing preserved bony margins with sclerotic changes around the lesion, consistent with a chronic slow‐growing process. Figure S2: Axial CT image illustrating posterior extension of the lesion within the frontal sinus. Figure S3: Axial T2‐weighted MRI image showing further extension of the lesion posteriorly. Figure S4: Axial T1‐weighted MRI showing the lesion as slightly hyperintense compared to brain parenchyma. Figure S5: Axial post‐contrast T1‐weighted MRI with gadolinium demonstrating no significant enhancement, consistent with a benign non‐vascular lesion. Figure S6: Axial gadolinium‐enhanced T1 MRI confirming absence of enhancement, supporting the diagnosis of an epidermoid cyst. Figure S7: Coronal T2 MRI showing detailed margins of the lesion and its relationship with the adjacent orbit. Figure S8: Coronal T2‐weighted MRI highlighting expansion of the right frontal sinus with thinning of the superior orbital wall. [file CCR3-13-e70887-s001.zip › Supplementary Figure 1.PNG]

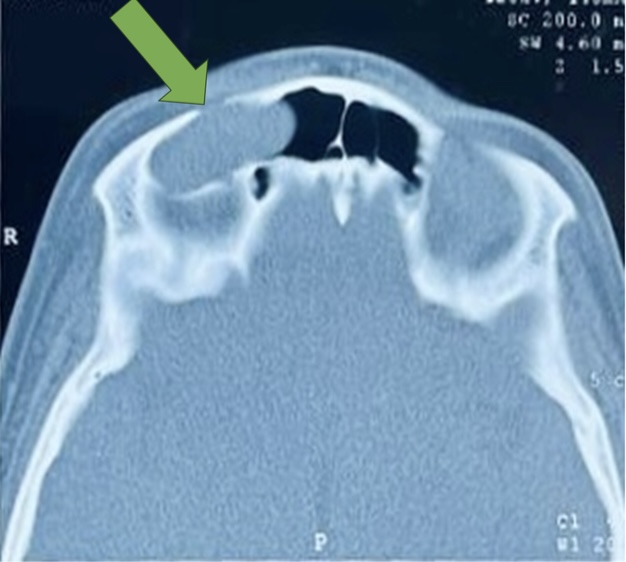

Supplement: Supplementary file 1 — Figure S1: Coronal CT view showing preserved bony margins with sclerotic changes around the lesion, consistent with a chronic slow‐growing process. Figure S2: Axial CT image illustrating posterior extension of the lesion within the frontal sinus. Figure S3: Axial T2‐weighted MRI image showing further extension of the lesion posteriorly. Figure S4: Axial T1‐weighted MRI showing the lesion as slightly hyperintense compared to brain parenchyma. Figure S5: Axial post‐contrast T1‐weighted MRI with gadolinium demonstrating no significant enhancement, consistent with a benign non‐vascular lesion. Figure S6: Axial gadolinium‐enhanced T1 MRI confirming absence of enhancement, supporting the diagnosis of an epidermoid cyst. Figure S7: Coronal T2 MRI showing detailed margins of the lesion and its relationship with the adjacent orbit. Figure S8: Coronal T2‐weighted MRI highlighting expansion of the right frontal sinus with thinning of the superior orbital wall. [file CCR3-13-e70887-s001.zip › Supplementary Figure 2.PNG]

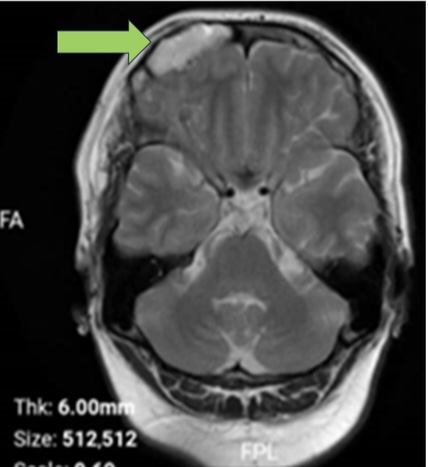

Supplement: Supplementary file 1 — Figure S1: Coronal CT view showing preserved bony margins with sclerotic changes around the lesion, consistent with a chronic slow‐growing process. Figure S2: Axial CT image illustrating posterior extension of the lesion within the frontal sinus. Figure S3: Axial T2‐weighted MRI image showing further extension of the lesion posteriorly. Figure S4: Axial T1‐weighted MRI showing the lesion as slightly hyperintense compared to brain parenchyma. Figure S5: Axial post‐contrast T1‐weighted MRI with gadolinium demonstrating no significant enhancement, consistent with a benign non‐vascular lesion. Figure S6: Axial gadolinium‐enhanced T1 MRI confirming absence of enhancement, supporting the diagnosis of an epidermoid cyst. Figure S7: Coronal T2 MRI showing detailed margins of the lesion and its relationship with the adjacent orbit. Figure S8: Coronal T2‐weighted MRI highlighting expansion of the right frontal sinus with thinning of the superior orbital wall. [file CCR3-13-e70887-s001.zip › Supplementary Figure 3.PNG]

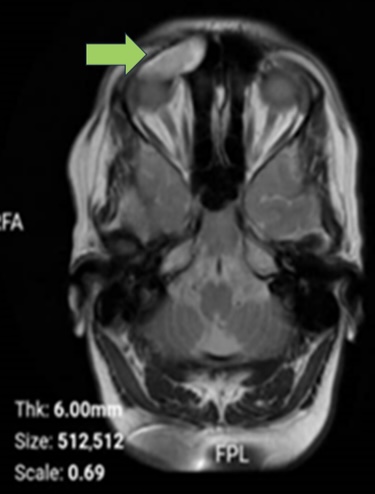

Supplement: Supplementary file 1 — Figure S1: Coronal CT view showing preserved bony margins with sclerotic changes around the lesion, consistent with a chronic slow‐growing process. Figure S2: Axial CT image illustrating posterior extension of the lesion within the frontal sinus. Figure S3: Axial T2‐weighted MRI image showing further extension of the lesion posteriorly. Figure S4: Axial T1‐weighted MRI showing the lesion as slightly hyperintense compared to brain parenchyma. Figure S5: Axial post‐contrast T1‐weighted MRI with gadolinium demonstrating no significant enhancement, consistent with a benign non‐vascular lesion. Figure S6: Axial gadolinium‐enhanced T1 MRI confirming absence of enhancement, supporting the diagnosis of an epidermoid cyst. Figure S7: Coronal T2 MRI showing detailed margins of the lesion and its relationship with the adjacent orbit. Figure S8: Coronal T2‐weighted MRI highlighting expansion of the right frontal sinus with thinning of the superior orbital wall. [file CCR3-13-e70887-s001.zip › Supplementary Figure 4.PNG]

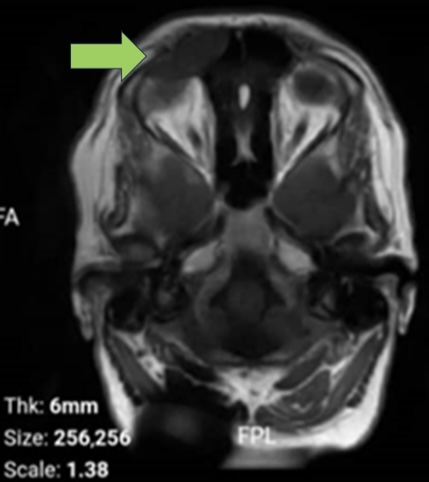

Supplement: Supplementary file 1 — Figure S1: Coronal CT view showing preserved bony margins with sclerotic changes around the lesion, consistent with a chronic slow‐growing process. Figure S2: Axial CT image illustrating posterior extension of the lesion within the frontal sinus. Figure S3: Axial T2‐weighted MRI image showing further extension of the lesion posteriorly. Figure S4: Axial T1‐weighted MRI showing the lesion as slightly hyperintense compared to brain parenchyma. Figure S5: Axial post‐contrast T1‐weighted MRI with gadolinium demonstrating no significant enhancement, consistent with a benign non‐vascular lesion. Figure S6: Axial gadolinium‐enhanced T1 MRI confirming absence of enhancement, supporting the diagnosis of an epidermoid cyst. Figure S7: Coronal T2 MRI showing detailed margins of the lesion and its relationship with the adjacent orbit. Figure S8: Coronal T2‐weighted MRI highlighting expansion of the right frontal sinus with thinning of the superior orbital wall. [file CCR3-13-e70887-s001.zip › Supplementary Figure 5.PNG]

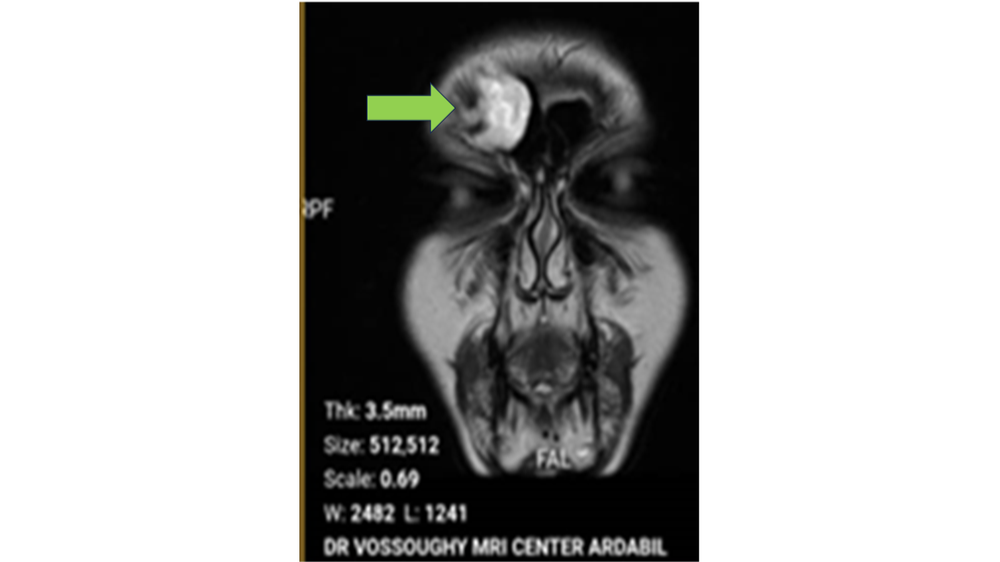

Supplement: Supplementary file 1 — Figure S1: Coronal CT view showing preserved bony margins with sclerotic changes around the lesion, consistent with a chronic slow‐growing process. Figure S2: Axial CT image illustrating posterior extension of the lesion within the frontal sinus. Figure S3: Axial T2‐weighted MRI image showing further extension of the lesion posteriorly. Figure S4: Axial T1‐weighted MRI showing the lesion as slightly hyperintense compared to brain parenchyma. Figure S5: Axial post‐contrast T1‐weighted MRI with gadolinium demonstrating no significant enhancement, consistent with a benign non‐vascular lesion. Figure S6: Axial gadolinium‐enhanced T1 MRI confirming absence of enhancement, supporting the diagnosis of an epidermoid cyst. Figure S7: Coronal T2 MRI showing detailed margins of the lesion and its relationship with the adjacent orbit. Figure S8: Coronal T2‐weighted MRI highlighting expansion of the right frontal sinus with thinning of the superior orbital wall. [file CCR3-13-e70887-s001.zip › Supplementary Figure 7.PNG]

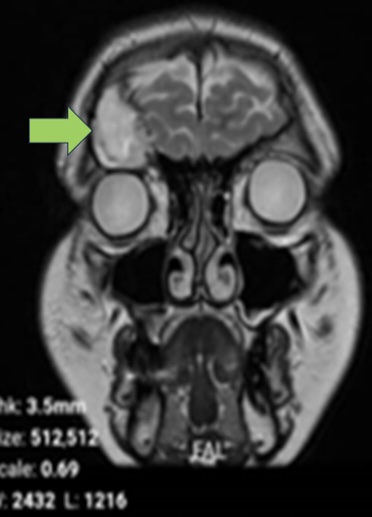

Supplement: Supplementary file 1 — Figure S1: Coronal CT view showing preserved bony margins with sclerotic changes around the lesion, consistent with a chronic slow‐growing process. Figure S2: Axial CT image illustrating posterior extension of the lesion within the frontal sinus. Figure S3: Axial T2‐weighted MRI image showing further extension of the lesion posteriorly. Figure S4: Axial T1‐weighted MRI showing the lesion as slightly hyperintense compared to brain parenchyma. Figure S5: Axial post‐contrast T1‐weighted MRI with gadolinium demonstrating no significant enhancement, consistent with a benign non‐vascular lesion. Figure S6: Axial gadolinium‐enhanced T1 MRI confirming absence of enhancement, supporting the diagnosis of an epidermoid cyst. Figure S7: Coronal T2 MRI showing detailed margins of the lesion and its relationship with the adjacent orbit. Figure S8: Coronal T2‐weighted MRI highlighting expansion of the right frontal sinus with thinning of the superior orbital wall. [file CCR3-13-e70887-s001.zip › Supplementary Figure 8.PNG]
